# Asymptomatic Apical Periodontitis Lesions and Their Association With Systemic Inflammatory Burden: A Preliminary Prospective Clinical Study

**DOI:** 10.7759/cureus.46357

**Published:** 2023-10-02

**Authors:** Kothandaraman Sathyanarayanan, N I Ranjana, Mohan Bhavana, Megavarnan R, Aravinthan Sankar, Selvakumar Mirnalini

**Affiliations:** 1 Conservative Dentistry and Endodontics, Sathyabama Dental College and Hospital, Chennai, IND

**Keywords:** inflammation, dental focal infection, innate immune response, chronic endodontic infection, apical periodontitis

## Abstract

Background*:* Apical periodontitis (AP) is an inflammatory disorder of the periapical tissues caused by the persistence of a microbial infection within the root canal system of the affected tooth. Clinically, it is symptomatic or asymptomatic depending on several factors such as the type of microorganisms, bacterial load, immunological reaction, and local tissue mediators. Chronic or asymptomatic infections may initiate and modulate intravascular accumulation of inflammatory cells resulting in endothelial dysfunction which subsequently represents a possible systemic inflammatory burden.

Aim*:* The present study aimed to evaluate the relationship between asymptomatic AP and systemic inflammatory burden by assessing the levels of chronic inflammatory cells.

Methodology: A total of 25 patients diagnosed with asymptomatic AP who showed a negative response to the pulp sensitivity test with no history of any systemic diseases were included in this preliminary prospective observational study. Blood samples were collected at each phase of the study, and a complete hemogram was carried out. All hematological parameters were recorded before and after root canal therapy and they were analyzed for statistical significance at p <.05 using IBM SPSS Statistics for Windows, Version 21 (Released 2012; IBM Corp., Armonk, New York, United States).

Results*:* Evaluation of the mean total leukocyte count (TLC), lymphocyte, and eosinophil cell count showed a significant reduction in the number of cells before and after root canal therapy treatment respectively (p<.05). One-way analysis of variance also revealed statistical significance at p < .05 with a weak positive correlation between the TLC, lymphocyte, and eosinophil count before and after treatment.

Conclusion*: *The present study showed that systemically healthy individuals with asymptomatic AP had increased inflammatory burden in the circulation, and thus, it is essential to identify and quantify the risk associated. It was evident that complete healing of the asymptomatic AP lesions results in an overall reduction of systemic inflammatory cells ultimately reducing the burden and risk of associated systemic inflammatory diseases.

## Introduction

Inflammation presents a complex biological protective response of body tissues to harmful stimuli such as microbes, chemical irritants, and injured or deceased cells with the involvement of immune cells, blood vessels, and molecular mediators. An extensive range of pathological stimuli can induce an inflammatory response in which pro-inflammatory cytokines and their receptors, cell adhesion molecules (CAM), cytokines, chemokine, and several chemical mediators of an intrinsic stress process participate in the healing of the injured cell or tissue [1]. Apical periodontitis (AP) is a microbial infection of the peri-radicular tissues characterized by a predominant inflammatory response within the root canal system of the affected tooth. AP often occurs after irreversible inflammation of the pulpal tissue and subsequent necrosis [2,3].

Inflammatory reactions of the peri-radicular tissue to numerous harmful stimuli were elicited by toxins, noxious byproducts, and degenerated pulp tissue rather than the pathogen itself. These irritants are capable of activating innate and adaptive immune responses either by non-antigenic or antigen-mediated pathways thus stimulating the production of acute or chronic inflammatory cells [4,5]. Studies have shown that asymptomatic peri-apical lesions may go undiagnosed over a period of time which subsequently leads to increased inflammatory cell load on the systemic circulation [6]. Another prospective case-control study also showed low-grade systemic inflammation resulting from inflammatory cells recruited by locally produced mediators that subsequently enter into the bloodstream and induce cell load [7]. Evidence from a recent systematic review and meta-analysis found a positive correlation between AP and elevated concentrations of inflammatory mediators associated with systemic inflammatory burden [8]. These findings indicate that there is an elevated systemic inflammatory cell in patients with chronic AP if left untreated.

The majority of these studies had largely focused on observing the relationship between local inflammatory cell load and AP, while an increase or decrease in the total leukocyte count (TLC) or differential leukocyte count as an indicator of systemic inflammatory burden following root canal therapy is left unexplored. Although studies have shown reduced clinical signs, symptoms, and decreased pro-inflammatory cytokine levels following root canal therapy, these observations lack a conclusion to elucidate the role of chronic inflammatory cells present in A on the systemic inflammatory burden and their influence of variation in systemic circulation for a patient with asymptomatic AP following root canal therapy. Hence, the present study aimed to evaluate the relationship between asymptomatic AP and inflammatory burden by assessing the circulatory levels of chronic inflammatory cells as an indicator of systemic inflammatory reaction.

## Materials and methods

The present clinical preliminary prospective study was carried out among patients between 18 and 40 years of age who reported to the outpatient Department of Conservative Dentistry and Endodontics, Sathyabama Dental College and Hospital, Chennai, India based on the following inclusion and exclusion criteria. The study included patients diagnosed with asymptomatic AP who showed a negative response to pulp sensitivity test with no history of any systemic diseases that included diabetes, hypertension, or any other inflammatory disorders, and showed significant radiographic lesions of size more than 3 mm under intra-oral periapical radiograph (IOPAR). Patients with known cases of systemic diseases, moderate to severe periodontal diseases, previously treated endodontic tooth with persistent AP, pregnancy, obesity, and with significant history of smoking, and alcohol were excluded.

A total of 25 participants were selected based on the above criteria and the study purpose was enlightened to all the participants followed by written informed consent to make certain of their voluntary participation. Ethical committee protocol approval was acquired from the institutional board ethical committee (EC/NEW/INST/2020/1397) and the study was done by the revised Helsinki Declaration of 2013. Root canal treatment was performed according to the standard endodontic procedure and all the sterilization protocols were strictly followed. Blood samples were collected before and after root canal treatment and a complete hemogram was carried out. All hematological parameters were recorded before and after root canal treatment and they were analyzed for statistical significance at p <.05 using IBM SPSS Statistics for Windows, Version 21 (Released 2012; IBM Corp., Armonk, New York, United States). A paired t-test was used for the comparison of variables within the groups and one-way analysis of variance (ANOVA) between the groups by using means was done for statistical significance at p <.05.

## Results

On analysis of the given data, the mean TLC before treatment was 8966.4 ± 1337.07 (Mean ± SD) cells per cu.mm (cubic millimeter), and the mean lymphocyte and eosinophil cell counts were observed as 28.84 ± 4.853 and 4.8 ± 1.384 (Mean ± SD) cells per cu.mm respectively. Evaluation after root canal therapy treatment showed that the mean TLC after treatment was 7918 ± 783.19 (mean ± SD) cells per cu.mm and the mean lymphocyte and eosinophil cell counts were observed as 24.96 ± 2.684 and 2.52 ± 0.7141 (mean ± SD) cells per cu.mm, respectively. On comparison of the above variable within the groups, a significant reduction in the number of cells before and after root canal therapy treatment was observed with the differences in the TLC, lymphocyte, and eosinophil count statistically significant at p<.05 (Table 1).

**Table 1:** Table showing the comparison of variables within the groups using the paired t-test * p<.05: Level of significance; S.D: standard deviation; Cu.mm: cubic millimeter.

Groups	Parameters	Mean ± SD (Cells per Cu. mm)	p-value*
Before Root Canal Therapy	Total Leukocyte Count	8966.4 ± 1337.07	.012*
	Lymphocyte	28.84 ± 4.853	
	Eosinophil	4.8 ± 1.384	
After Root Canal Therapy	Total Leukocyte Count	7918 ± 783.19	.0005*
	Lymphocyte	24.96 ± 2.684	
	Eosinophil	2.52 ± 0.7141	

One-way ANOVA of the mean TLC before and after treatment revealed an F-ratio (Fisher) of 11.44388 with a p-value of .001436 statistical significance at p < .05 (Table 2). Similarly, one-way ANOVA of the mean lymphocyte cell count before and after treatment revealed an F-ratio (Fisher) of 12.234 with a p-value of .00102 statistical significance at p < .05 (Table 3). One-way ANOVA of the mean eosinophil count before and after treatment revealed a higher F-ratio (Fisher) of 53.559 with a p-value of .00001 statistical significance at p < .05 (Table 4). The overall F-ratio (Fisher) revealed a weak positive correlation observed between the study group variance and the within-group variance.

**Table 2:** Table showing the one-way analysis of variance (ANOVA) between the groups by using means of total leukocyte count (TLC) * p<.05: Level of significance; S.D: standard deviation; N: total samples

Groups	N	Mean	S.D	p-value
Before Root Canal Therapy	25	8966.4	1337.07	.0014*
After Root Canal Therapy	25	7910	783.19	

**Table 3:** Table showing the one-way analysis of variance (ANOVA) between the groups by using means of lymphocyte count * p<.05: Level of significance; S.D: standard deviation; N: total samples

Groups	N	Mean	S.D	p-value
Before Root Canal Therapy	25	28.84	4.853	.0010*
After Root Canal Therapy	25	24.96	2.684	

**Table 4:** Table showing the one-way analysis of variance (ANOVA) between the groups by using means of eosinophil count * p<.05: Level of significance; S.D: standard deviation; N: total samples

Groups	N	Mean	S.D	p-value
Before Root Canal Therapy	25	4.8	1.3844	.00001*
After Root Canal Therapy	25	2.52	0.7141	

## Discussion

AP is characterized by the presence of infectious and inflammatory responses by the host tissue and immunological cells. Depending on the state of infection, it can be either acute or chronic disease capable of inducing specific and non-specific immune responses in the periapical region. Literature studies have shown that chronic infection is characterized by the presence of inflammatory cell infiltrate predominantly mononuclear cells where T-cells, macrophages, and neutrophils assist the formation of AP [9]. In the present study, comparing the study variables within the groups (Group I and Group II) showed a significant increase in the number of cells before root canal treatment with the differences in TLC, lymphocyte, and eosinophil count. These results are in accordance with previous observations that have shown the major cells involved in chronic inflammatory reactions are mononuclear phagocytes, B- and T-lymphocytes, neutrophils, and fibroblasts [9,10]. It was also evident that chronic or asymptomatic infections may initiate and modulate intravascular accumulation of inflammatory cells resulting in endothelial dysfunction which may subsequently represent a possible systemic inflammatory burden [11-13].

Zhang et al. investigated the role of AP in the systemic elevation of inflammatory markers and pathological changes in distant organs in an animal model and reported clinical evidence that AP may trigger a systemic immune response with a subsequent increase in the cell count [11]. Sirin et al. studied the association between C-reactive protein, neutrophil-to-lymphocyte ratio, and the burden of AP and correlated it with the new proposed scoring system for AP [5]. It was observed that root canal treatment performed in individuals with AP had a reduced postoperative reduction in levels of pro-inflammatory cytokines. Similarly, the present study also revealed a significant reduction in the levels of TLC, lymphocyte, and eosinophil count before and after root canal therapy treatment with a statistical difference at p<.05 suggestive of a possible association between chronic AP and circulatory systemic inflammatory cells. These results and the previous observations suggest that AP is associated with a generalized increase in systemic inflammatory cells and associated mediators like C-reactive protein, IL-1, IL-2, IL-6, and immunoglobulin levels compared to healthy individuals.

The exact mechanism for the reduction of eosinophil remains unclear nonetheless Graunaite et al. observed that in the early phase of inflammation, cytokines induce chemoattractant for monocytes and other cells at the site of infection thus contributing to the increased or decreased circulatory cells in the bloodstream [10]. Silva et al. noted migration of lymphocytes and polymorphonuclear cells induces the release of chemical mediators responsible for cell recruitment at the site of apical infection and activation of other inflammatory cells such as eosinophils, plasma cells, and mast cells thus possibly promoting the cells to enter the bloodstream [12]. A recent prospective case-control study by Georgiou et al. also showed low-grade systemic inflammation resulting from inflammatory cells recruited by locally produced mediators that subsequently enter into the bloodstream to induce cell load [7]. Many interventional studies have been performed to evaluate the serum levels of inflammatory markers in patients with AP before and after various treatments and reported significant reductions after treatment [13,14]. van der Waal noted a weak association between chronic inflammatory biomarkers and low-grade systemic inflammation in patients with AP [15]. Similarly in the present study, a weak positive correlation was observed between the lymphocytes and AP before and after root canal therapy. Evidence from a systematic review and meta-analysis by Gomes et al. and Márton et al. also found a positive correlation between AP and elevated concentrations of inflammatory mediators associated with systemic inflammatory burden supporting the present results [8,16].

Recommendations

Further studies and research work with a larger sample size and a long-term follow-up period are necessary to confirm the relationship between asymptomatic AP and systemic inflammatory burden.

Limitations

The present study largely focused on the chronic inflammatory cells; however, assessing the presence of specific inflammatory biomarkers and their levels in serum levels are needed to validate the current evidence that chronic AP can influence the systemic inflammatory state. Similar clinical studies are further needed on a larger scale supported by immunological and immunohistochemical analysis on the circulatory inflammatory markers following root canal therapy to support the assumption.

## Conclusions

Periapical lesions develop as a result of interaction between bacteria, their byproducts, and the host defense mechanism. The present study showed systemically healthy individuals with asymptomatic AP had increased inflammatory burden in the circulation, and thus, it is essential to identify and quantify the risk associated. Thus, it is evident that thorough debridement of the infected root canal systems followed by complete healing of the asymptomatic AP lesions results in an overall reduction of systemic inflammatory cells, ultimately reducing the burden and risk of associated systemic inflammatory diseases.
